# Frequency and characterization of ancillary chest CT findings in COVID-19 pneumonia

**DOI:** 10.1259/bjr.20200716

**Published:** 2021-01-20

**Authors:** Mario Silva, Roberta Eufrasia Ledda, Mark Schiebler, Maurizio Balbi, Sandro Sironi, Francesca Milone, Paola Affanni, Gianluca Milanese, Nicola Sverzellati

**Affiliations:** 1 Department of Medicine and Surgery, Unit of “Scienze Radiologiche”, University of Parma, Parma, Italy; 2 Department of Radiology, UW-Madison School of Medicine and Public Health, Madison, WI, USA; 3 Department of Radiology, ASST Papa Giovanni XXIII, University of Milano-Bicocca, Milan, Italy; 4 Laboratorio di Igiene e Sanità Pubblica, Dipartimento di Medicina e Chirurgia, Università di Parma, Parma, Italy

## Abstract

**Objectives:**

Ground-glass opacity and consolidation are recognized typical features of Coronavirus disease-19 (COVID-19) pneumonia on Chest CT, yet ancillary findings have not been fully described. We aimed to describe ancillary findings of COVID-19 pneumonia on CT, to define their prevalence, and investigate their association with clinical data.

**Methods:**

We retrospectively reviewed our CT chest cases with coupled reverse transcriptase polymerase chain reaction (rt-PCR). Patients with negative rt-PCR or without admission chest CT were excluded. Ancillary findings included: vessel enlargement, subpleural curvilinear lines, dependent subpleural atelectasis, centrilobular solid nodules, pleural and/or pericardial effusions, enlarged mediastinal lymph nodes. Continuous data were expressed as median and 95% confidence interval (95% CI) and tested by Mann–Whitney *U* test.

**Results:**

Ancillary findings were represented by 106/252 (42.1%, 36.1 to 48.2) vessel enlargement, 50/252 (19.8%, 15.4 to 25.2) subpleural curvilinear lines, 26/252 (10.1%, 7.1 to 14.7) dependent subpleural atelectasis, 15/252 (5.9%, 3.6 to 9.6) pleural effusion, 15/252 (5.9%, 3.6 to 9.6) mediastinal lymph nodes enlargement, 13/252 (5.2%, 3 to 8.6) centrilobular solid nodules, and 6/252 (2.4%, 1.1 to 5.1) pericardial effusion. Air space disease was more extensive in patients with vessel enlargement or centrilobular solid nodules (*p* < 0.001). Vessel enlargement was associated with longer history of fever (*p* = 0.035) and lower admission oxygen saturation (*p* = 0.014); dependent subpleural atelectasis with lower oxygen saturation (*p* < 0.001) and higher respiratory rate (*p* < 0.001); mediastinal lymph nodes with shorter history of cough (*p* = 0.046); centrilobular solid nodules with lower prevalence of cough (*p* = 0.023), lower oxygen saturation (*p* < 0.001), and higher respiratory rate (*p* = 0.032), and pericardial effusion with shorter history of cough (*p* = 0.015). Ancillary findings associated with longer hospital stay were subpleural curvilinear lines (*p* = 0.02), whereas centrilobular solid nodules were associated with higher rate of intensive care unit admission (*p* = 0.01).

**Conclusion:**

Typical high-resolution CT findings of COVID-19 pneumonia are frequently associated with ancillary findings that variably associate with disease extent, clinical parameters, and disease severity.

**Advances in knowledge:**

Ancillary findings might reflect the broad range of heterogeneous mechanisms in severe acute respiratory syndrome from viral pneumonia, and potentially help disease phenotyping.

## Introduction

The diagnosis of severe acute respiratory syndrome Coronavirus 2 (SARS-CoV-2) infection is confirmed by viral nucleic acid detection.^1,2^ The infection from SARS-CoV-2 with clinical impairment is known as coronavirus disease 2019 (COVID-19) and this is frequently represented by pulmonary damage up to acute respiratory distress syndrome (ARDS), which is termed COVID-19 pneumonia and recognized as a major cause of mortality.^3^

The role of radiology in managing COVID-19 has been evaluated since the earliest Chinese outbreak of SARS-CoV-2 and recommendations were thereafter issued by International scientific societies.^4–7^ The Fleischner Society recommended the use of chest imaging in patients with confirmed COVID-19 infection and worsening respiratory status, and in those with suspected infection presenting with moderate-to-severe clinical features in resource-constrained environments. They also highlighted the higher sensitivity of chest CT over chest radiography for early parenchymal lung abnormalities, disease progression, and alternative diagnoses.^3^ High-resolution CT (HRCT) has been largely performed to assist the clinical evaluation of patients with suspected COVID-19 pneumonia.^6–9^ This widespread use of HRCT led to definition of typical HRCT findings of COVID-19 pneumonia, which are described as patchy peripheral ground-glass opacity (GGO) with or without consolidation.^2,10–13^ Moreover, a number of less frequent findings such as centrilobular solid nodules, intrapulmonary vessels enlargement, subpleural curvilinear lines, pleural or pericardial effusion have been reported in some series, however without full characterization.^14^ A detailed characterization of these findings might contribute in integrated clinicoradiological stratification of disease severity.

The purpose of this study was to describe ancillary findings on HRCT of patients with laboratory confirmed COVID-19 pneumonia, to define their prevalence, and to test their association with clinical data and in-hospital clinical deterioration.

## Methods and materials

### Ethics statement

This study was approved by the local Institutional Review Board and informed consent was waived.

### Study population

**Figure 1.**
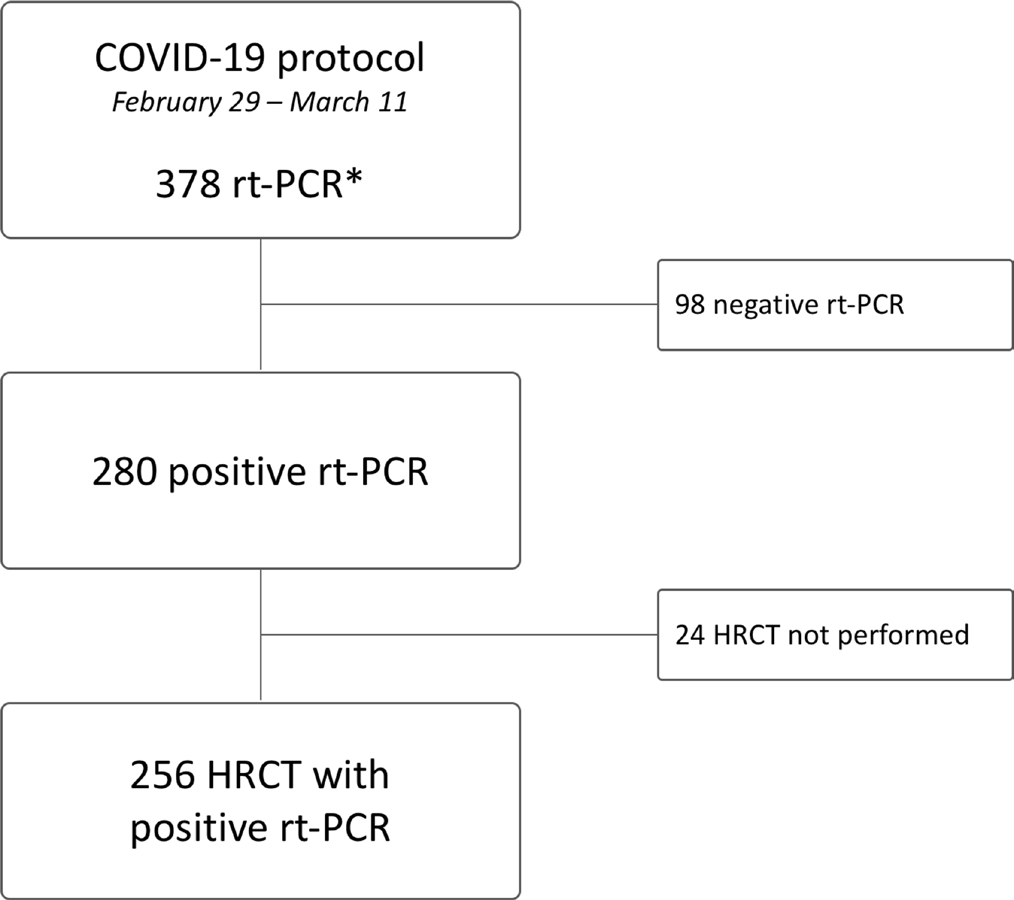
Flow-chart of patient selection according to rt-PCR availability and positive outcome. *rt-PCR outcome was collected on first swab for the present study. HRCT, high-resolution CT; rt-PCR, reverse transcription polymerase chain reaction.

378 consecutive patients referred to the respiratory triage between February 29 and March 11, 2020. Patients were screened for symptoms: temperature >37.5°C, oxygen saturation <95%, respiratory rate >25 breaths per minute, and/or history of recent cough.^15^ Provided clinical evaluation of respiratory status, patients with moderate to severe pulmonary involvement underwent HRCT scan. Subjects with at least one positive rt-PCR and chest HRCT on admission were included in this study. Patients with negative rt-PCR or without admission HRCT were excluded (Figure 1). Clinical data were obtained from medical records.

### Imaging technique

Non-contrast HRCT was performed with either a 128-slice scanner (SOMATOM Definition Edge, Siemens Healthineers, Erlangen, Germany) or a 16-slice mobile scanner on truck (SOMATOM Emotion, Siemens Healthineers, Erlangen, Germany). HRCT images were acquired with the patient in the supine position during end-inspiration breath-hold. The acquisition parameters were 100–140 kVp on the 128-row scanner (automatic selection of tube voltage by CareKv, Siemens Healthineers) or fixed 110 kVp on the 16-row scanner, 80 reference mAs, pitch 1.0–1.5, and collimation 0.625–1.0 mm. Reconstruction parameters for lung images: slice thickness 1.0 mm, increment 0.7–1.0 mm, sharp reconstruction algorithm (Bl57 or B70s, respectively), lung window (width, 1600 Hounsfield unit, HU; level, −600 HU). Reconstruction parameters for mediastinal images: slice thickness 2.0 mm, increment 1.5 mm, medium reconstruction algorithm (Br36 or B31s, respectively), mediastinal window (width, 400 HU; level, 30 HU). Advanced Modeled iterative reconstruction (ADMIRE) strength 3 on the 128-row scanner, filtered back projection (FBP) on the 16-row scanner.

### Imaging interpretation

A chest radiologist with 17 years of experience (NS) in chest imaging retrospectively reviewed the HRCT scans on the local Picture Archiving and Communication System (PACS) workstation (suite Estensa, Esaote, Genova, Italy). The study reader was aware that the study population included only patients with confirmed diagnosis of SARS-CoV-2 infection.

Typical findings: the extent of combined GGO and consolidation was visually scored at the nearest 5% on the whole lungs. The distribution was described as follows*:* (a) axial distribution: predominantly peripheral (within the outer third of the lung), predominantly central, or mixed; (b) craniocaudal distribution: predominantly upper (above the carina), middle (between the carina and the right inferior pulmonary vein) or lower (below the right inferior pulmonary vein)^16^ ; (c) bilateral or unilateral involvement; (d) lobar involvement was accounted over six lobes (lingula was considered as a single lobe). Description of the pattern was also tabulated into the categories of our local COVID-19 protocol.^15^ These categories aimed to define disease severity by encompassing both morphology and extent of parenchymal findings, as follows: (1) non-COVID-19 findings, (2) findings indeterminate for COVID-19, either because of differential or overlapping disease, (3) typical pattern of COVID-19, including different combinations of GGO and consolidations and their overall extent (Table 1 and Supplementary Figure 1).^15^ In particular, category 2 included any HRCT with findings that did not suggest exclusive alternative diagnosis nor isolated COVID-19 pneumonia, and it was therefore meant either as “differential between diseases” or “overlapping diseases”. For instance, category 2 as “differential between diseases” (COVID-19 or other disease) was assigned when GGO (with or without consolidation) were seen with a pattern compatible with other diseases (*e.g.* pulmonary edema). Otherwise, category 2 as “overlapping diseases” (COVID-19 and other disease) was assigned when HRCT findings typical for other diseases (*e.g.* fibrotic lung disease, oncologic disease, bacterial infection) were seen in association with typical findings of COVID-19 pneumonia. Category 3 was defined by the appearance of HRCT pattern typical for COVID-19 (GGO with or without consolidation, with multifocal–multilobar distribution and peripheral predominance), and it was further described across a range of disease severity that included both morphology (exudative *vs* organized morphology) and extent by visual score.

Supplementary Figure 1.

**Table 1.** Summary categories prospectively used in Parma University Hospital for assisting clinical decision during high-flow phase of COVID-19 epidemic

		Extent	N^o^	% (95% CI)	Relative extent median (95% CI)
Category 1	Normal		0/256	0%	–
	Non-COVID disease – Report alternative diagnosis		4/256	1.6% (0.6% –3.9%)	40% (30%–65%)^*a*^
Category 2	Indeterminate for COVID				
	Differential diagnosis COVID-19 OR other disorders		14/256	5.5% (3.3% –9.0%)	20% (15%–30%)
	Up to three focal abnormalities (up to about 3–4 cm in max diameter)	Mild	3/256	1.2% (0.4% –3.4%)	10% (5%–15%)^a^
	More than three focal abnormalities (above 3–4 cm in max diameter)	Moderate/severe	11/256	4.3% (2.4% –7.5%)	25% (15–35%)
	Suspected overlap COVID-19 AND other disorders		25/256	9.8% (6.7% –14.0%)	35% (20%–40%)
	Up to three focal abnormalities (up to about 3–4 cm in max diameter)	Mild	3/256	1.2% (0.4%–3.4%)	10% (5%–15%)^*a*^
	More than three focal abnormalities (above 3–4 cm in max diameter)	Moderate/severe	22/256	8.6% (5.7%–12.7%)	35% (20%–40%)
Category 3	Typical				
	Pure patchy ground-glass opacities		107/256	41.8% (35.9% –47.9%)	30% (20%–40%)
	Up to three focal abnormalities (up to about 3–4 cm in max diameter)	Mild	11/256	4.3% (2.4%–7.5%)	10% (5%–15%)
	More than three focal abnormalities (above 3–4 cm in max diameter)	Moderate/severe	96/256	37.5% (31.8% –43.6%)	30% (20%–40%)
	Focal ground-glass opacities admixed with “early” consolidation		27/256	10.5% (7.4%–14.9%)	20% (15%–45%)
	Up to three focal abnormalities (up to about 3–4 cm in max diameter)	Moderate/severe	4/256	1.6% (0.6%–3.9%)	10% (5%–15%)*^a^*
	*More than three focal abnormalities (above 3–4 cm in max diameter*)	Moderate/severe	23/256	8.9% (6.1%–13.1%)	15% (10%–45%)
	Diffuse ground-glass opacities (distribution may be heterogeneous)	Severe	27/256	10.5% (7.4%–14.9%)	20% (15%–40%)
	Ground-glass admixed with perilobular opacities or consolidation with signs of distortion^*b*^	Severe	52/256	20.3% (15.8% –25.7%)	30% (20%–40%)

CI, confidence interval.

a Differential between “moderate” and “severe” is entirely subjective and will not impact on the decision about hospitalization.

b This category was chosen in the presence of conspicuous organized consolidation, despite predominant pattern was still ground-glass.

The retrospective expert reading was compared with the prospective clinical reading from clinical practice (general radiologists with experience range 3–30 years), the interobserver agreement was tested for category and extent of disease.

Ancillary findings: additional HRCT findings reported as “ancillary findings” included the following: enlarged intrapulmonary vessel within GGO, subpleural curvilinear lines, dependent subpleural atelectasis, pleural effusion, mediastinal lymph node enlargement, centrilobular solid nodules, and pericardial effusion.

**Figure 2.**
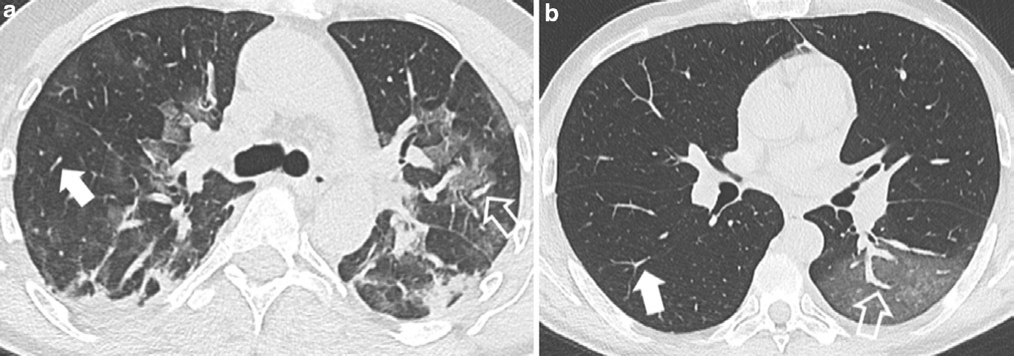
(**a, b**) Axial CT image without contrast shows enlarged pulmonary artery within ground-glass opacity (open arrow in a) compared with homologous vessel in the contralateral lung (solid arrow in a). Pulmonary vein within patchy area of ground glass opacity (open arrow in b) appears substantially enlarged compared with homologous vessel in the contralateral lung (solid arrow in b).

*Enlarged vessel within the areas of GGO* was qualitatively assessed on the axial plane, by comparing the “enlarged vessel” with homologous contralateral vascular structures passing through normal parenchyma (Figure 2). When the contralateral area of lung parenchyma was also affected, the comparison was made with vascular structures at the same distance from the pleura surface passing through normal parenchyma, in the same slice. The “enlarged vessel” sign was classified as appearing in dependent and non-dependent regions of the lung or both. Pulmonary vessels were differentiated into pulmonary arteries or veins by tracking the vessel centrally to the mediastinum.

*Subpleural curvilinear lines* were defined as thin curvilinear opacity, 1–3 mm in thickness, lying less than 1 cm from and parallel to the pleural surface.

**Figure 3.**
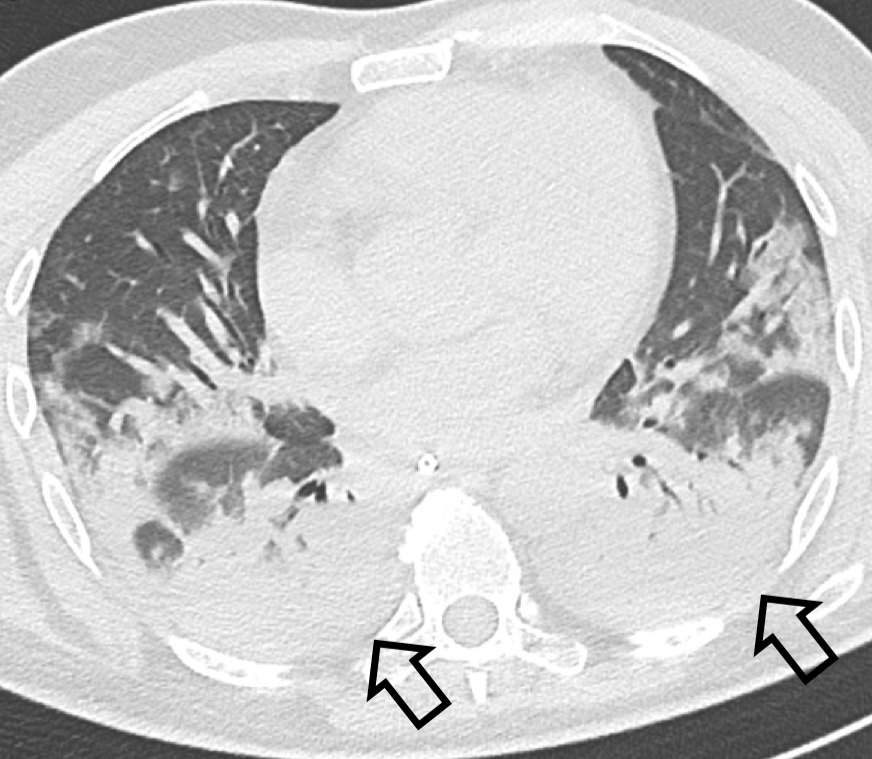
Axial CT image without contrast showing dependent subpleural atelectasis (open arrows).

Dependent subpleural atelectasis was described as consolidation with dependent crescent shape (Figure 3).

*Pleural effusion* was defined by pleural fluid thickness ≥5 mm measured on the axial plane with the mediastinal window at the level of maximum thickness of the pleural fluid; the side of effusion was also recorded.

*Mediastinal lymph nodes* with a short-axis diameter >10 mm were deemed significantly enlarged. The number of enlarged mediastinal lymph nodes was recorded along with the node station.

**Figure 4.**
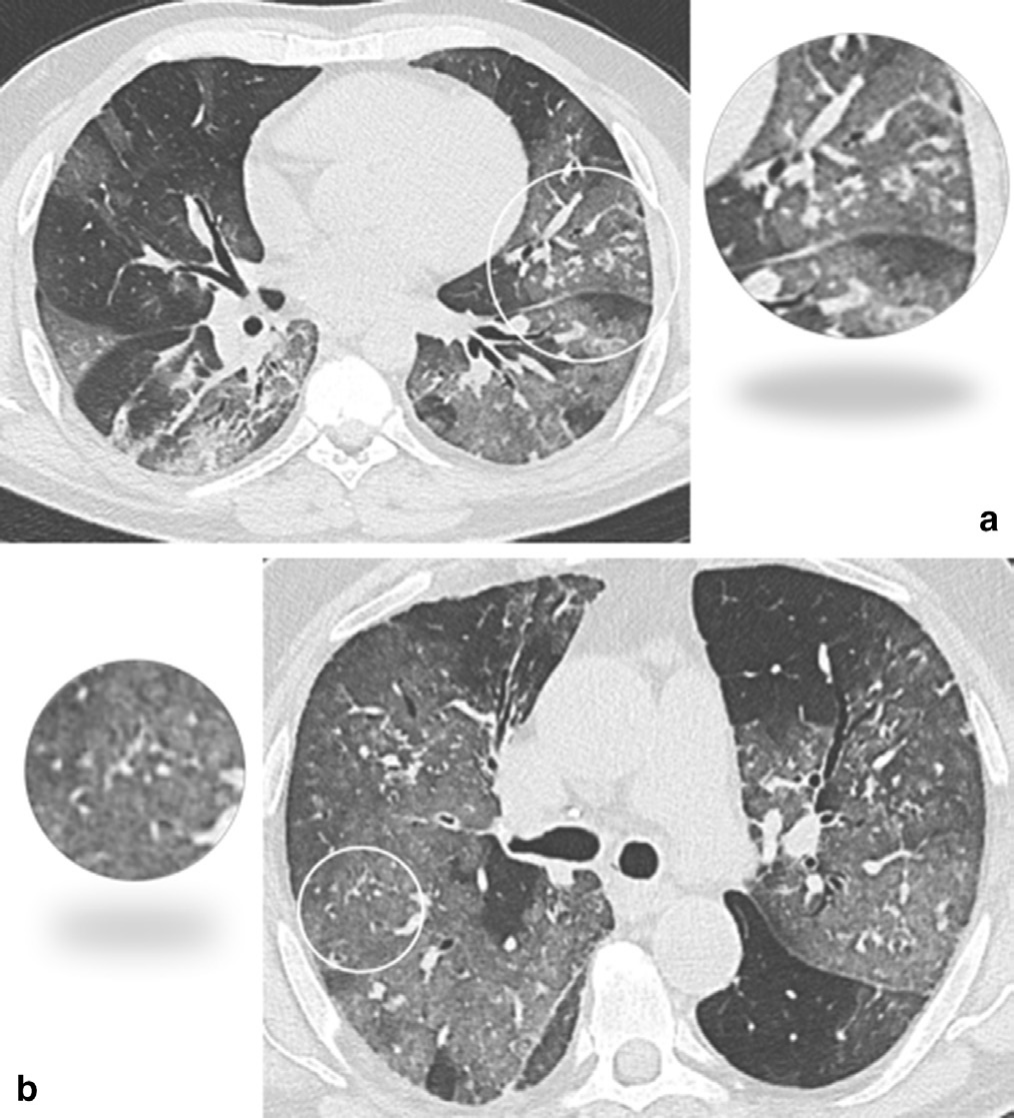
(**a, b**) Axial CT image without contrast showing solid centrilobular nodules (open circle and magnified vignette).

*Centrilobular solid nodules* were defined by size <10 mm (by measuring the largest recognizable nodule in the lungs for each patient) (Figure 4) and shape (free text was allowed for the description). The adjacency to vessels was also recorded.

*Pericardial effusion* was measured at the maximal circumferential extent of the pericardium at four points around the circumference: anterior, posterior, left lateral, and right lateral pericardium.^17^ The sum of these four measurements was then calculated.

All terms were defined in accordance with Fleischner Society glossary.^18^

### Statistical analysis

Continuous data were expressed as median and its 95% confidence interval (95% CI) and tested by Mann–Whitney *U* test. Categorical data were expressed as absolute and relative distribution, with corresponding 95% CI using Wilson method, and were tested using the Fisher exact test. Interobserver agreement was tested by Cohen’s K test with quadratic weights (*k_w_*) and its 95% CI. A *p*-value < 0.05 was deemed statistically significant. Statistical analysis was performed by MedCalc Software bvba (v. 19.1–64-bit, Ostend, Belgium).

## Results

### Patient demographics and clinical data

A total of 256/280 (91.4%, 87.6–94.2%) patients (158 males and 98 females, median age 71 years, range 32–98) were included in this study (Figure 1). 215/256 (83.9%, 78.9–87.9%) patients reported a history of fever prior to admission (median days 6, 95% CI 5–7) as compared to a median temperature measured on admission 37.1°C (95% CI 36.9–37.3°C); 137/256 (53.5%, 47.4–59.5%) patients presented with cough (median days 6, 95% CI 5–7), median respiratory rate was 22 breaths per minute (95% CI 20–24), and median oxygen saturation was 95% (95% CI 94–95%), notably with five patients (2.1%, 0.9–4.9%) who had already been on oxygen therapy at the time of admission.

Arterial hypertension (40.6%, 34.8–46.7%), malignancy (11.3%, 8–15.8%), and ischaemic heart disease (10.5%, 7.3–14.9%) were the most represented comorbidities.

The patients were admitted to temporary wards until the result of rt-PCR (median stay 8 day, 7–9). 20 patients (7.8%, 5.1–11.7%) required ICU admission and 6 (2.3%, 1.1–5%) were still in hospital at the time of the observation.

### Typical findings

A total of 252/256 (98.4%, 96.1–99.4%) HRCT scans showed typical findings of COVID-19 pneumonia: 159/256 (62.1%, 56–67.8%) GGO, either patchy or diffuse; 37/256 (14.4%, 10.7–19.3%) mixed GGO and consolidation; 52/256 (20.3%, 15.8–25.7%) GGO admixed with organized consolidation; 4/256 (1.6%, 0.6–3.9%) multifocal consolidations. HRCT showed consolidations suggestive of bacterial pneumonia in the remainder 4/256 (1.6%, 0.6–3.9%) patients. The frequency of each predefined radiological category is reported in Table 1; interobserver agreement was moderate for disease morphology (k_w_ = 0.59, 0.49–0.70) and excellent for disease extent (k_w_ = 0.87, 0.84–0.91).

Lung opacities showed mostly mixed axial distribution (149/252; 59.1%, 53.0–65.0%), without craniocaudal predominance (173/252; 68.7%, 62.7–74.1%), bilateral (245/252; 97.2%, 94.4–98.7), and with all lobes involved (185/252; 73.4%, 67.6–78.5%) (Table 2).

**Table 2.** Distribution of typical findings in COVID-19 pneumonia

Distribution of typical findings in COVID-19 pneumonia		N°	% (95% CI)	Extent median (95% CI)
Axial distribution	Peripheral	100/252	39.7% (33.8%–45.8%)	15% (15%–20%)
	Central	3/252	1.2% (0.4%–3.4%)	25%
	Mixed	149/252	59.1% (53.0%–65.0%)	40% (35%–45%)
Craniocaudal distribution	Upper	5/252	2% (0.8%–4.5%)	5% (5%–30%)
	Middle	16/252	6.3% (4.0%–10.1%)	25% (15%–30%)
	Lower	58/252	23% (18.3%–28.6%)	15% (15%–20%)
	No predominance	173/252	68.7% (62.7%–74.1%)	35% (30%–40%)
Bilateral involvement	245/252		97.2% (94.4%–98.7%)	30% (25%–35%)
Number of lobes involved	1	8/252	3.2% (1.6%–6.1%)	5% (5%–15%)
	2	9/252	3.6% (1.9%–6.7%)	5% (5%–10%)
	3	13/252	5.2% (3.0%–8.6%)	5% (5%–15%)
	4	10/252	3.9% (2.2%–7.2%)	10% (5%–20%)
	5	27/252	10.7% (7.4%–14.9%)	15% (15%–20%)
	6	185/252	73.4% (67.6%–78.5%)	40% (35%–40%)

CI, confidence interval.

Typical findings-only were seen in 84/252 (33.3%, 27.8–39.3%) patients as opposed to 168/252 (66.7%, 60.6–72.3%) patients showing both typical and ancillary findings.

### Ancillary findings

Ancillary findings were represented by 106/252 (42.1%, 36.1–48.2%) vessel enlargement, 50/252 (19.8%, 15.4–25.2%) subpleural curvilinear lines, 26/252 (10.1%, 7.1–14.7%) dependent subpleural atelectasis, 15/252 (5.9%, 3.6–9.6%) pleural effusion, 15/252 (5.9%, 3.6–9.6%) mediastinal lymph nodes enlargement, 13/252 (5.2%, 3–8.6%) centrilobular solid nodules, and 6/252 (2.4%, 1.1–5.1%) pericardial effusion. Combinations of multiple ancillary findings were observed in 49/252 (19.2%, 15–24.8%) patients: 2 ancillary findings in 42/252 (16.4%, 12.6–21.8%) patients, 3 in 5/252 (2%, 0.9–4.6%), and 4 in 2/252 (0.8%, 0.2–2.8%).

The overall extent of HRCT findings (including typical and ancillary findings) was higher in patients with enlarged vessel (40%, 35–45%) compared to those without (20%, 15–20%; *p* < 0.001), and in patients with centrilobular solid nodules (65%, 55–75%) compared to those without (25%, 20–30%; *p* < 0.001) (Table 3).

**Table 3.** Overall extent of disease according to presence of ancillary findings

	With ancillary finding	Without ancillary finding	*p*
	(*n* = number of patients) median (95% CI)	(*n* = number of patients) median (95% CI)	
Vessel enlargement	(*n* = 106)	(*n* = 146)	<0.001
	40% (35%–45%)	20% (15%–20%)	
Subpleural curvilinear lines	(*n* = 50)	(*n* = 202)	0.943
	30% (20%–40%)	30% (20%–35%)	
Dependent subpleural atelectasis	(*n* = 26)	(*n* = 226)	0.449
	30% (20%–40%)	25% (20%–35%)	
Pleural effusion [12 mm (95% CI 6 to 47)]	(*n* = 15)	(*n* = 237)	0.073
	45% (25%–70%)	30% (20%–35%)	
Mediastinal lymph node enlargement	(*n* = 15)	(*n* = 237)	0.659
	30% (15%–40%)	30% (20%–35%)	
Centrilobular solid nodules	(*n* = 13)	(*n* = 239)	<0.001
	65% (55%–75%)	25% (20%–30%)	
Pericardial effusion [18 mm (95% CI 13 to 109)]	(*n* = 6)	(*n* = 246)	0.205
	15% (10%–40%)	30% (20%–35%)	

CI, confidence interval.

Features of ancillary findings are thereafter detailed:

- Enlarged vessels within GGO areas were both arteries and veins in 81/106 (76.4%, 67.5–83.5%) patients, only veins in 17/106 (16%, 10.3–24.2%), and only arteries in 8/106 (7.6%, 3.9–14.2%). Among the 98/106 (92.4%, 85.8–96.1%) patients with veins enlargement, both dependent and non-dependent distribution was seen in 65/98 (66.3%, 56.5–74.9%) cases, dependent in 20/98 (20.4%, 13.6–29.4%), and non-dependent in 13/98 (13.3%, 7.9–21.4%). Among the 89/106 (83%, 75.8–89.7%) cases with enlarged arteries, both dependent and non-dependent distribution was seen in 43/89 (48.3%, 38.2–58.5%), non-dependent in 27/89 (30.3%, 21.8–40.5%), and dependent 19/89 (21.4%, 14.1–31%).
- Enlarged vessel were associated with patchy GGO in 73/106 (68.9%, 59.5–76.9%) patients and diffuse GGO in 33/106 (31.1%, 23.1–40.5%).
- Subpleural curvilinear lines were limited to the lower lobes, associated with both GGO and consolidation in 27/50 (54%, 40–67%), patchy GGO in 18/50 (36%, 24.1–49.9%), and diffuse GGO 5/50 (10%, 4.4–21.4%).
- Dependent subpleural atelectasis was always limited to the lower lobes, associated with both GGO and consolidation in 22/26 (84.6%, 66.5–93.9%), patchy GGO in 2/26 (7.7%, 2.1–24.1%), diffuse GGO in 2/26 (7.7%, 2.1–24.1%). Association with subpleural curvilinear lines was seen in 3/26 (11.5%, 4–29%) patients.
- Pleural effusion was bilateral in 10/15 (66.7%, 41.7–84.8%) patients, unilateral right in 3/15 (20%, 7.1–45.2%), unilateral left in 2/15 (13.3%, 3.7–37.9%); median thickness was 12 mm (6-47). Pleural effusion was associated with diffuse GGO in 9/15 (60%, 35.8–80.2%) patients, both GGO and consolidation in 3/15 (20%, 7.1–45.2%), only patchy GGO in 2/15 (13.3%, 3.7–37.9%), and only consolidation in 1/15 (6.7%, 1.2–29.8%).
- The median number of enlarged mediastinal lymph nodes was 4^3,4,19^ ; the most represented stations were 7 (13 lymph nodes in 10 patients) and 4R (24 lymph nodes in 12 patients). Enlarged mediastinal lymph nodes were associated with patchy GGO in 7/15 (46.7%, 24.8–69.9%) patients, both GGO and consolidation in 7/15 (46.7%, 24.8–69.9%), and only consolidation in 1/15 (6.6%, 1.2–29.8%).
- Centrilobular solid nodules were mostly polyhedral in shape (10/13, 76.9%, 49.7–91.8%). Only one case showed tree-in-bud morphology because of nodules attached to small vessels. Their maximal diameter was approximately 10 mm. Nodules were exclusively admixed with GGO, notably associated with diffuse GGO in 9/13 (69.2%, 42.4–87.3%) patients, patchy GGO in 2/13 (15.4%, 4.3–42.2%), and GGO and consolidation in 2/13 (15.4%, 4.3–42.2%).
- Pericardial effusion showed a median thickness of 18 mm (13-109), it was associated with diffuse GGO in 2/6 (33.3%, 9.7–70%) patients, patchy GGO in 2/6 (33.3%, 9.7–70%), and both GGO and consolidation in 2/6 (33.3%, 9.7–70%).

### Ancillary findings and clinical data

Ancillary findings and symptoms are detailed in Table 4. Vessel enlargement was associated with longer history of fever (*p* = 0.035) and lower admission oxygen saturation (*p* = 0.014). Overall, dependent subpleural atelectasis was associated with lower oxygen saturation (*p* < 0.001) and higher respiratory rate (*p* < 0.001). Fourteen out of 26 (53.9%, 35.5–71.3%) patients with subpleural atelectasis showed an overall pulmonary involvement ≤30%.^12^ In this subgroup the median oxygen saturation was 90% (87–94%) *vs* 89% (81–91%; *p* = 0.0.278) of the remaining 12 patients with an overall extent >30%; the median respiratory rate was 25 in both subgroups (95% CI 24–25 and 25–28, respectively; *p* = 0.563). Mediastinal lymph nodes were associated with shorter history of cough (*p* = 0.046). Centrilobular solid nodules were associated with lower prevalence of cough (*p* = 0.023), lower oxygen saturation (*p* < 0.001), and higher respiratory rate (*p* = 0.032).

**Table 4.** Distribution of clinical parameters according to ancillary findings

	Days with fever	*p*	*p*	Cough	*p*	Days with cough	*p*	O2 Saturation	*p*	Respiratory rate	*p*
	Median (95% CI)			Median (95% CI)		Median (95% CI)		Median (95% CI)		Median (95% CI)	
Vessel enlargement									0.014	22 (20–24)	0.987
*No (n = 146*)	6 (5–6)	0.035	0.957	53.4% (45.3–61.3)	0.797	6 (5–7)	0.462	95 (94– 95)			
*Yes (n = 106*)	7 (6–7)			55.7% (46.1–64.7)		7 (5–7)		94 (92–95)		22 (20–24)	
Subpleural curvilinear lines									0.725	22 (20–24)	0.778
*No (n = 50*)	6 (6–7)	0.338	0.852	11.4% (7.7–16.5)	1	6 (5–7)	0.151	95 (94–95)			
*Yes (n = 202*)	7 (6–7)			54% (40.4–67)		7 (6–8)		95 (93–95)		23 (21–25)	
Dependent subpleural atelectasis									<0.001	22 (20–23)	<0.001
*No (n = 26*)	6 (6–7)	0.213	0.699	54.4% (47.9–60.7)	1	6 (5–7)	0.449	95 (94–95)			
*Yes (n = 226*)	6 (3–7)			53.8% (35.4–71.2)		5.5 (3–7)		89 (85–92)		25 (22–28)	
Pleural effusion									0.149	22 (20–23)	0.078
*No (n = 15*)	6 (6–7)	0.074	0.990	55.3% (48.9–61.4)	0.291	6 (5–7)	0.678	95 (94–95)			
*Yes (n = 237*)	5 (3–6–)			40% (19.8–64.2)		4.5 (2–14)		92 (84–96)			
Mediastinal lymph node enlargement									0.339	22 (20–24)	0.374
*No (n = 15*)	6 (6–7)	0.371	0.498	54.8% (48.4–61.1)	0.599	6 (6–7)	0.046	95 (94–95)			
*Yes (n = 237*)	4 (3–9)			46.6% (24.8–69.8)		3 (2–7)		91 (90–95)		24 (20–28)	
Centrilobular solid nodules									<0.001	22 (20–24)	0.032
*No (n = 13*)	6 (6–7)	0.676	0.885	56.1% (49.7–62.2)	0.023	6 (5–7)	0.964	95 (94–95)			
*Yes (n = 239*)	6 (3–7)			23.1% (8.2–50.3)		7 (NA)		86 (80–92)		30 (18–41)	
Pericardial effusion									0.766	22 (20–24)	0.266
*No (n = 6*)	6 (6–7)	0.104	0.551	54.5% (48.2–60.6)	1	6 (6–7)	0.015	95 (94–95)			
*Yes (n = 246*)	3 (NA)			50% (18.7–81.2)		2 (NA)		95 (NA)		26 (NA)	

CI, confidence interval.

We did not observe association between ancillary findings and comorbidities (Table 5).

**Table 5.** Distribution of cardiovascular, respiratory and oncological comorbidities in according to presence of any ancillary finding

Comorbidity	Overall	With ancillary findings	Typical findings-only	*p*
	N°/256 %	N°/252 %	N°/252 %	
	(95% CI)	(95% CI)	(95% CI)	
Arterial hypertension	104	67	36	0.684
	40.6%	26.6%	14.3%	
	(34.8%–46.7%)	(21.5%–32.3%)	(10.5%–19.1%)	
Malignancy	29	20	8	0.673
	11.3%	7.9%	3.2%	
	(8%–15.8%)	(5.2%–11.9%)	(1.6%–6.1%)	
**Ischemic heart disease**	27	20	7	0.517
	10.5%	7.9%	2.8%	
	(7.3%–14.9%)	(5.2%–11.9%)	(1.3%–5.6%)	
Atrial fibrillation	26	20	6	0.279
	10.1%	7.9%	2.4%	
	(7%–14.4%)	(5.2%–11.9%)	(1.1%–5%)	
COPD	21	16	5	0.469
	8.2%	6.3%	1.9%	
	(5.4%–12.2%)	(3.9%–10%)	(0.8%–4.5%)	
Asthma	11	5	6	0.187
	4.3%	1.9%	2.4%	
	(2.4%–7.5%)	(0.8%–4.5%)	(1.1%–5%)	
Pulmonary fibrosis	5	3	2	1
	1.9%	1.2%	0.8%	
	[0.8%–4.4%]	(0.4%–3.4%)	(0.2%–2.8%)	

CI, confidence interval.

Ancillary findings associated with in-hospital disease severity were subpleural curvilinear lines (longer hospital stay, *p* = 0.02) and centrilobular solid nodules (higher rate of ICU admission, *p* = 0.01) (Table 6).

**Table 6.** Correlation between days of hospitalization, ICU admission and ancillary findings

	Days of hospitalization	*p*	ICU admission	*p*
	Median (95% CI)		Median (95% CI)	
Vessel enlargement				
No (n = 146)	8 (7–9)	0.701	5.5% (2.8–10.4)	0.155
Yes (n = 106)	7 (6–9)		10.4% (5.9–17.6)	
Subpleural curvilinear lines				
No (n = 50)	7 (6–8)	0.02	6.4% (3.8–10.7)	0.227
Yes (n = 202)	8.5 (7–11)		12% (5.6–23.8)	
Dependent subpleural atelectasis				
No (n = 26)	8 (7–8)	0.921	7% (4–11.1)	0.426
Yes (n = 226)	9 [4–11)		11.5% (4–28.9)	
Pleural effusion				
No (n = 15)	8 (7–9)	0.449	7.2% (4.5–11.1)	0.314
Yes (n = 237)	8 (6–14)		13.3% (3.7–37.8)	
Mediastinal lymph node enlargement				
* No (n = 15)*	*8 (7–8.5)*	0.904	8% (5.2–12.2)	–
Yes (n = 237)	7 (4–13)		0% (NA)	
Centrilobular solid nodules				
No (n = 13)	8 (7–9)		6.2% (3.8–10.1)	
Yes (n = 239)	6 (2–12)	0.493	30.7% (12.6–57.6)	0.01
Pericardial effusion				
No (n = 6)	8 (7–8)	0.173	7.7% (5–11.7)	*–*
Yes (n = 246)	11.5 (6–5)		0% (NA)	

CI, confidence interval.

## Discussion

We report ancillary findings in 42.1% of patients with COVID-19 pneumonia. Vessel enlargement and centrilobular solid nodules were found in patients with more extensive pulmonary involvement. The presence of vessel enlargement, centrilobular solid nodules and dependent subpleural atelectasis was associated with a worse clinical status on admission. Moreover, centrilobular solid nodules were associated with a higher rate of ICU admission.

Recognized typical findings of COVID-19 pneumonia are GGO and consolidation.^10,12,18,20^ Such typical presentation can be variably associated with less common findings, which might be termed “ancillary findings”. Some ancillary findings were previously mentioned in the literature, while others are still underdescribed.

We proposed a methodical approach to define enlarged intrapulmonary vessels, namely by side-by-side-comparison (Figure 2). Enlarged intrapulmonary vessels were previously described in 45.2–82.4% of patients with COVID-19 pneumonia.^6,10,20–23^ Of note, the variability in reporting this sign might depend on its relatively subtle appearance, which is prone to interpretation. These HRCT features are observed both in dependent and non-dependent regions, which makes it different from the typically dependent vascular redistribution commonly observed in cardiac failure.^24^ Because vessel enlargement was seen only in GGO areas, it could be hypothesized that it represents a local response to local damage.^24–26^ Unfortunately, we did not have sufficient laboratory data to investigate association of this finding with coagulopathy.

Consolidation reported in COVID-19 pneumonia included exudative morphology as well as signs of organized pneumonia (OP) with pulmonary distortion.^27^ We are keen on describing a further type of consolidation in COVID-19 pneumonia, namely dependent subpleural atelectasis with specific morphology as opposed to clearly infectious pneumonia (Figure 3). Previous authors interpreted this finding in COVID-19 pneumonia as fibrosis or edema, without pathologic confirmation.^14^ Dependent atelectasis is well known in ICU in patients with severe respiratory decay and need for advanced respiratory support.^28^ On admission chest CT, we observed dependent subpleural atelectasis in association with lower oxygen saturation and increased respiratory rate, even in those patients with pneumonia extent ≤30%. This type of consolidation might represent dependent atelectasis deriving from damage alveolar epithelium and representing functional alveolar dead space, already described in ARDS.^29^ Progressive recruitment of these alveolar units can be assisted by prone positioning,^30^ which has been proved to be effective in COVID-19 patients, both conscious and treated with non-invasive ventilation,^31^ and critically ill who required intubation and invasive ventilation.^32^ Although it remains speculative, the specific finding of subpleural atelectasis on admission CT might contribute in treatment choice and serve as an indicator of a potentially worse clinical course.

Prior studies including more than 100 patients reported centrilobular solid nodules in 3% of patients with COVID-19 pneumonia,^31,33^ whereas smaller series described a much higher prevalence (up to 27.3%)^9,22,32^ ; however, there is insufficient description of specific features for differential with small airway disease.^32,33^ We described solid centrilobular nodules with polyhedral shape in 5.2% of patients with COVID-19 pneumonia (Figure 4). This morphology is different from the rounded or branching centrilobular nodules observed in small airway disease.^18^ Such detailed characterization might help in the differential with other infections, however there are practical limits from subjective interpretation and from supervening bacterial co-infection^34^ or other viral pneumonia.^35^ ^34,36^ We observed this finding always close to enlarged vessels within GGO, opening to speculation about associated vasculopathy.^37^ In our series, this finding was associated with disease severity at admission and during hospital stay. Aware of the limited representation of this finding in our population, we believe that its detection ought to prevent any delay in medical treatment.

We report subpleural curvilinear lines in 19.8% of patients and observed a positive association between their presence and longer hospitalization. Similar findings are typically described in subjects with exposure to asbestos.^38^ In up to 50% of cases, it was found to be associated with areas of consolidations, which had been correlated with more severe outcomes in ARDS patients.^39^

Finally, only a minority of patients presented with mediastinal lymph node enlargement, pleural or pericardial effusion, in line with prior investigations.^7,10,21,37–41^ These relatively non-specific findings within a complex pathology (*e.g.* involvement of both pulmonary and cardiac failure) hampers the possibility to draw hypothesis on such a small sample size.

Our study has several limitations. First, the retrospective design, is prone to confounding factors such as selection of patients; however, we tried to limit this bias by selecting consecutive rt-PCR positive subjects. Second, survival data were not available nor definitely certified for a substantial proportion of this population, and thus not included in the analysis, limiting our possibilities to investigate their prognostic relevance. Third, clinical information was not available for all patients enrolled, affecting the significance of the attempted correlation between ancillary findings and clinical picture. Further investigation of these findings is warranted by comprehensive inclusion of clinical parameters as well as with comparison against subjects with negative rt-PCR. Fourth, the presence of a single reader limited the objectiveness of interpretation of ancillary findings and does not warrant on the repeatability of these data.

In conclusion, typical HRCT findings of COVID-19 pneumonia are quite frequently associated with ancillary findings that variably associate with disease extent, clinical parameters, and disease severity. Correlation with survival data and follow-up CTs are fostered for in-depth understanding of ancillary findings and their clinical relevance.
