# Supplementary material for: Frequency and characterization of ancillary chest CT findings in COVID-19 pneumonia
Source: Br J Radiol. 2021 Jan 20;94(1118):20200716. doi: 10.1259/bjr.20200716 (PMC7934290; doi:10.1259/bjr.20200716)

Supplementary figures

## Supplementary Figure 1: category 2 differential.

Consolidations in right upper lobe and lingula, patchy ground glass opacities (GGO), and bronchial wall thickening in the upper lobes. The association of multifocal consolidations and GGO appears highly suspicious for COVID-19 pneumonia, however the bronchial wall thickening suggests airway disease (e.g. bronchopneumonia).


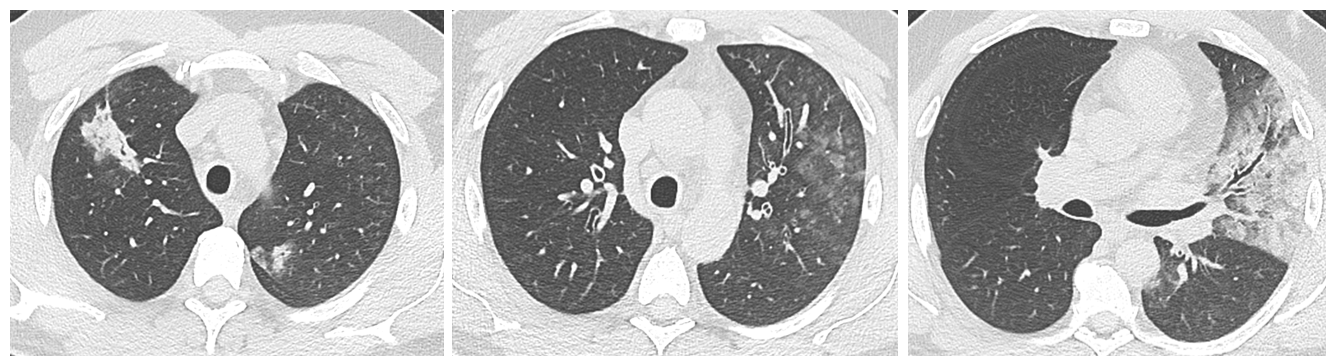


## Supplementary Figure 2: category 2 overlapping.

Diffuse ground glass opacities overlaying chronic fibrotic changes. The rapid onset of diffuse GGO during the SARS-CoV-2 epidemic suggests COVID-19 pneumonia overlapping chronic fibrotic lung disease.


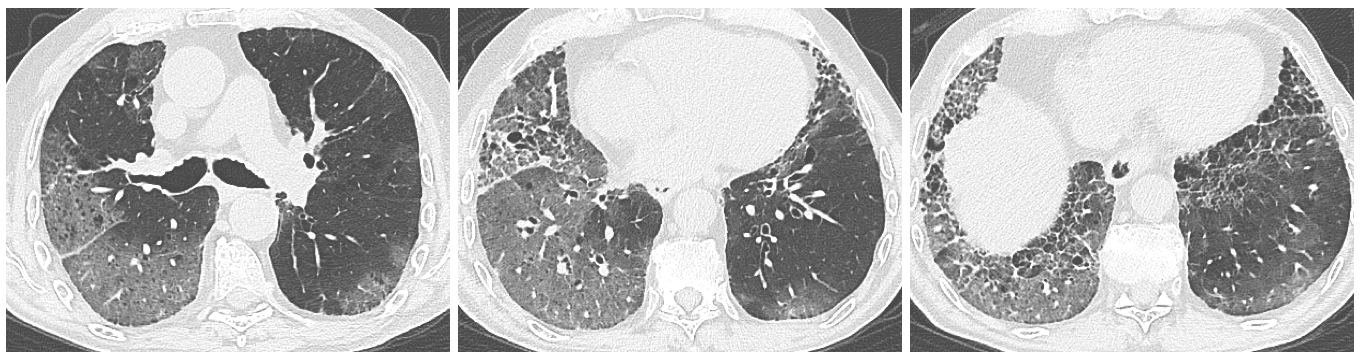


## Supplementary Figure 3: category 3 patchy GGO.

Ground glass opacities with multifocal, bilateral, and patchy distribution into one potential presentation of COVID-19 pneumonia.


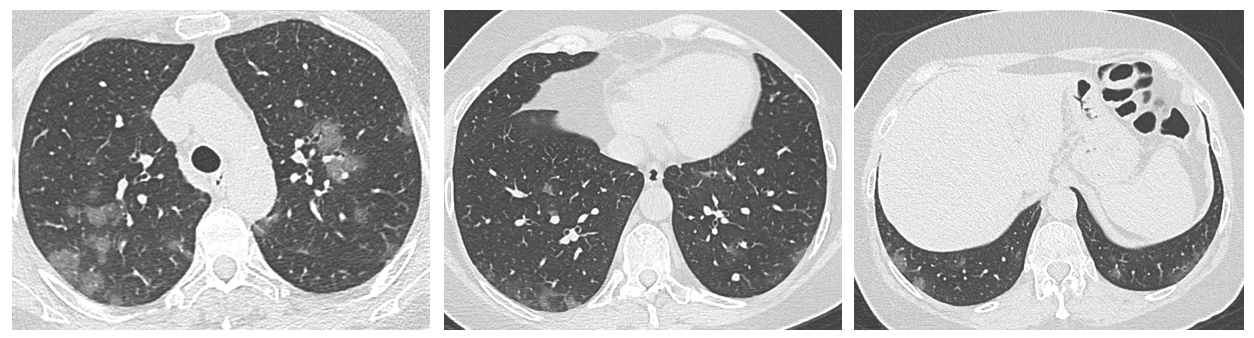


## Supplementary Figure 4: category 3 diffuse GGO.

Diffuse bilateral ground glass opacities into one possible HRCT appearance of COVID-19 pneumonia.


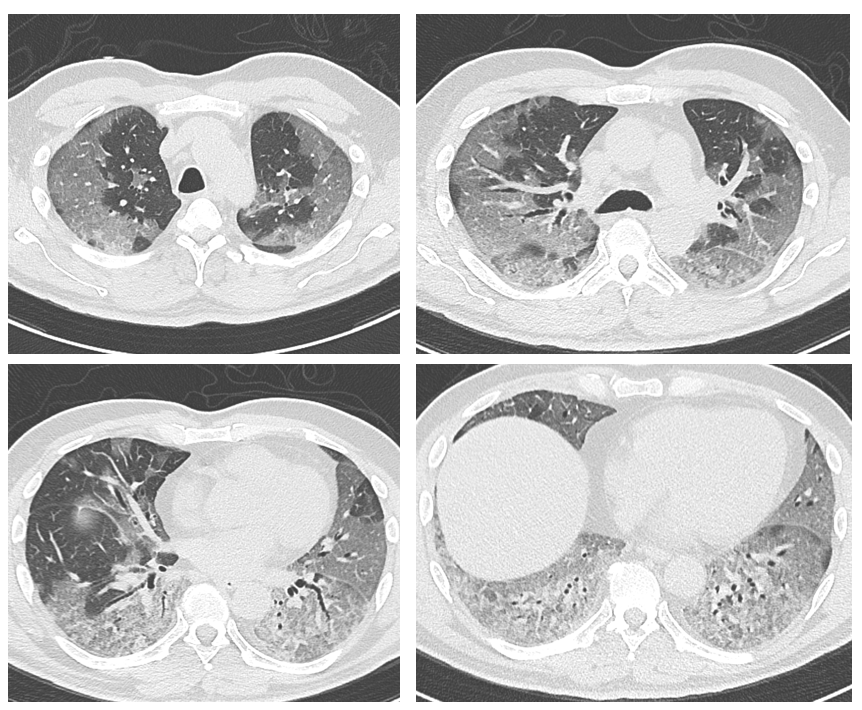


## Supplementary Figure 5: category 3 GGO and organized consolidations.

Ground glass opacities with multifocal, bilateral, subpleural, and patchy distribution associate with consolidations and signs of parenchymal distortion into one possible CT appearance of COVID-19 pneumonia, potentially in a later phase with foci organizing pneumonia.


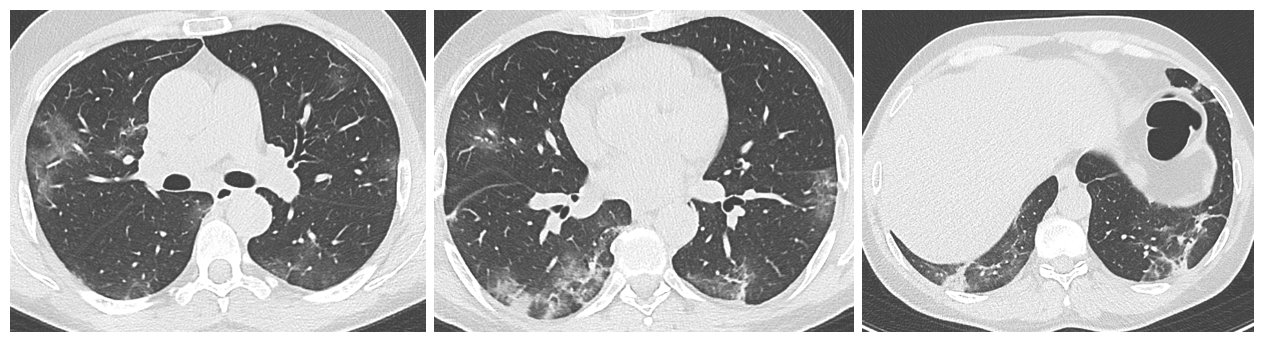

Supplement: Supplementary Figure 1. [file bjr.20200716.suppl-01.docx]
